# Why ‘understanding’ of research may not be necessary for ethical emergency research

**DOI:** 10.1186/s13010-020-00090-7

**Published:** 2020-08-26

**Authors:** Dan Kabonge Kaye

**Affiliations:** College of Health Sciences, Department of Obstetrics and Gynecology, Makerere University, P.O. Box 7072, Kampala, Uganda

## Abstract

**Background:**

Randomized controlled trials (RCTs) are central to generating knowledge about effectiveness of interventions as well as risk, protective and prognostic factors related to diseases in emergency newborn care. Whether prospective participants understand the purpose of research, and what they perceive as the influence of the context on their understanding of the informed consent process for RCTs in emergency obstetric and newborn care are not well documented.

**Methods:**

Conceptual review.

**Discussion:**

Research is necessary to identify how the illnesses may be prevented, to explore the causes, and to investigate what medications could be used to manage such illness. Voluntary informed consent requires that prospective participants understand the disclose information about the research, and use this to make autonomous informed decision about participation, in line with their preferences and values. Yet the emergency context affects how information may be disclosed to prospective research participants, how much participants may comprehend, and how participants may express their voluntary decision to participate, all of which pose a threat to the validity of the informed consent. I challenge the claim that the ‘understanding’ of research is always necessary for ethical informed consent for research during emergency care. I argue for reconceptualization of the value of understanding, through recognition of other values that may be equally important. I then present a reflective perspective that frames moral reflection about autonomy, beneficence and justice in research in emergency research.

**Conclusion:**

While participant ‘understanding’ of research is important, it is neither necessary nor sufficient for a valid informed consent, and may compete with other values with which it needs to be considered.

## Background

In order to participate in randomized clinical trials, prospective participants are required to provide informed consent for participation. On invitation to participate, information regarding the study is disclosed to them or their caregivers so that they make a voluntary informed decision about whether or not to participate, depending on their preferences and values [1, 2]. The International Conference on Harmonization [3] defines informed consent as *“the process by which an individual voluntarily expresses his or her willingness to participate in a particular trial, after having been informed of all aspects of the trial that are relevant to the decision to participate”.* In emergency obstetric care research, the informed consent process requires that potential participants comprehend the disclosed information, which, among other essentials, includes the purpose of the research, the key study procedures, potential benefits, potential risks and alternatives to participation, so they can weigh the potential risks against potential benefits and make an informed voluntary decision ICH [3]. Often, in emergency research contexts, the consent process may not enable detailed explanations to enable understanding of the clinical trial [4–6]. In the current best practice, written trial information often describes what will happen during clinical trials, however, it is debatable whether participants comprehend the information as some aspects of the trial may not be understood by participants [5, 6]. Also, why participants are expected to behave in a certain manner, or why participants must accept that certain procedures have to be performed in a stipulated manner, may not be clear to participants [5, 6]. Consequently, potential participants may create their own interpretations, and make the *apparently* informed decision to consent, refuse to participate or withdraw from research when they are perceived to be inadequately informed [5, 6]. In this paper, I use ‘understanding’ to refer to the process of using the disclosed information to make informed decisions whether or not to participate or continue to participate in research, after due consideration of potential risks, benefits and alternatives. Whether potential research participants should ‘understand’ research in the context of emergency obstetric care, in spite of the many influences that constrain understanding, is the basis of the argument in this paper.

## Main text

### Obligations to seek informed consent for research participation

The intrinsic value of understanding research is enshrined in international codes and regulations. The Nuremburg code [7] requires that for research, *“voluntary consent is essential” and “the results of any experiment must be for the greater good of society”.* The Belmont Report [8] defined three basic ethical principles for biomedical research: Respect for Persons, beneficence and justice, which are related to informed consent. For informed consent to be valid, the Belmont Report [8] requires that there is disclosure of (1) sufficient relevant information for decision making, (2) comprehension (of the disclosed information), and (3) voluntariness. That is, for valid informed consent, the participant should receive sufficient relevant information, should adequately understand the information provided, and should arrive at a decision without being subjected to coercion or undue influence.

The obligation to seek voluntary informed consent emanates from the ethical principle of respect for autonomy. Beauchamp and Childress [1] emphasize this moral vale that *“respect for autonomy does provide the primary justification of rules, policies, and practices of informed consent*.” They assert that autonomy refers to *“autonomous action in terms of normal choosers who act intentionally, … with understanding … , and … without controlling influences that determine their action”.* Therefore, valid informed consent requires the researcher to provide potential participants with detailed information about what research participation requires, determine that the potential participant understands the disclosed information, and ensure that the consent provided is voluntary [1]. In their perspective [1], personal autonomy *encompasses, at a minimum, self-rule that is free from both controlling interference by others and from certain limitations such as an inadequate understanding that prevents meaningful choice. The autonomous individual acts freely in accordance with a self-chosen plan. …* *.* Thus, viewed from a negative perspective, personal autonomy is freedom from others’ control and undue influence, while positively speaking, it is choosing (and subsequently acting) in accordance with one’s *“self-chosen plan.”* From this perspective the obligation of the researchers in respecting autonomy is *“to respect autonomous agents … to acknowledge their right to hold views, to make choices, and to take actions based on their personal values and beliefs”.* Dean [9] stresses personal autonomy as *“the power to make decisions in accord with one’s overall ends and plans”* and presents the rationale of disclosing information about the research as *“to allow patients to make choices that fit with their overall desires, goals, and attitudes” and as the “intuitive force of the principle of respect for autonomy.”* Beauchamp and Childress [1] emphasize that autonomous choice is an imperative, (even if it may be illogical or irrational) as they suggest that (… *to respect autonomous agents is to acknowledge their right to hold views, to make choices, and to take actions based on their personal values and beliefs).*

### The goal of the informed consent

The informed consent for clinical research has as its goals both to respect and promote a participant’s autonomy and to protect participants from potential harms [10–13]. A valid informed consent process requires that the following concepts are present: disclosure, understanding, capacity, voluntariness and decision [1, 14, 15,]. Disclosure involves giving research participants all relevant information about the research, including its nature, purpose, risks and potential benefits as well as the alternatives available [14]. Understanding implies that research participants are able to comprehend the information provided and appreciate its relevance to their personal situations [1, 15]. Capacity relates to an individual’s ability to make decisions that stems from his or her ability to understand the information provided [15]. Voluntariness means that an individual’s decision to participate is made without coercion or persuasion [1, 14, 15]. Providing informed consent to participate in clinical trials is a critical pillar of the ethical conduct of research, which necessitates providing *adequate information* to a *competent decision-maker* through a process that ensures a *voluntary decision process* [1, 14–16]. How voluntariness is conceptualized and measured varies in different studies [16].

The International Conference on Harmonization of Technical Requirements for Registration of Pharmaceuticals for Human Use (ICH) Good Clinical Practice Guidelines (GCP) [3] defines informed consent as *“a process by which a subject voluntarily confirms his or her own willingness to participate in a particular trial after having been informed of all aspects of the trial that are relevant to the subject’s decision to participate.”* Thus the ICH Good Clinical Practice (GCP) guidelines [3] indicate that whereas it is the responsibility of researchers to develop an informed consent form (ICF) that includes sufficient relevant information to enable a potential research participant’s decision making, it is the role of the Institutional Review Board (IRB) or Independent Ethics Committee (IEC) to review both the ICF and the informed consent process or procedures to ensure the obtainment of valid consent [Ref]. As such it becomes essential, in the pursuit of valid consent, that there is understanding of the concepts underlying the essential ICF elements required by internationally recognized guidelines and regulations. The validity of the informed consent in clinical research is determined by the extent to which participants understand the process of informed consent [17], as comprehension directly affects how ethical principles are applied in practice [18–20]. The extent to which participants understand research is variable [21–23], calling into question whether participant ‘understanding’ is always necessary for research.

### Participants “understanding” of the research and quality of informed consent

The Declaration of Helsinki provides ethical standards that should govern clinical research to ensure respect for human subjects, protection of their health and rights, and to minimize the possibility of exploitation [2]. The informed consent process is deemed to have the following requirements in order to be considered valid: disclosure of information, comprehension, voluntariness, capacity to make a decision and finally the decision to participate [24]. That is, in the context of emergency obstetric care, prospective participants should understand the diagnosis, prognosis, nature and purpose of the intervention, alternatives, potential risks, and benefits. In practice, the consenting physician or researcher should endeavor to convey the information to the fullest extent, both orally and/or in writing, in a manner and language which is appropriate and tailored to each individual’s level of understanding [3, 25]. However, there are numerous shortcomings which have been documented regarding participants’ understanding of consent process, including what they consent to, to such an extent that the informed consent process is often reduced to a simple recitation of the contents of the written document. The systematic reviews [26, 27] conducted on consent assessment studies show that certain elements of informed consent, such as randomization, the experimental nature of a study, availability of alternative treatments, distinguishing study and non-study procedures and compensation for trial-related injuries are universally difficult to grasp for participants.

The quality of informed consent in clinical research is determined by the extent to which the research participants understand the process and different elements of informed consent [28]. However, systematic reviews have shown variations in the extent and nature of participants’ understanding of different components of informed consent. For instance, Tam et al. [26] in a review of 103 studies, showed that about 60–70% of participants understand the components regarding purpose of the study, potential risks and adverse effects, confidentiality, availability of alternative treatments, and knowledge about comparability of treatments. Also, Mandava et al [27] found that contrary to the assumption, understanding of different elements of informed consent varied markedly in both developing and developed countries. The lack of understanding ranges from lack of awareness about participation in research to poor understanding of specific elements (such as experimental and therapeutic aspects of clinical trials) [29].

### Assessment of participants’ ‘understanding’ of research

The conceptualization of autonomous choices [1] considered *“normal choosers who act intentionally, with understanding … and without controlling influence that determine their action*”. Therefore, assessment of ‘understanding’ should consider adjustments for whether choosers are ‘normal’, whether they act intentionally’, and whether controlling influences that determine the choice are absent or relatively insignificant. While there is an imperative to assess participant understanding of research in general and the informed consent process in particular, participant ‘understanding’ is variable. Self-completion questionnaires are a useful tool for assessment as they are shorter, easier to follow, cheaper and quicker to administer, providing an advantage in a clinical trial setting where participants are already burdened by the trial requirements on their visits [30]. The Quality of Informed Consent (QuIC) is a brief, reliable, and valid questionnaire developed to assess the subject’s grasp of important general concepts about clinical trials concepts [29, 31].

The extent of lack of ‘understanding’ of research is high. For instance, a systematic review found that only around 50% of participants understood the components of informed consent process in surgical and clinical trials [21]. Two reviews [22, 23] noted variability in the theoretical conceptualization of the informed consent process elements. Besides, a multi-center, cross-sectional, descriptive survey conducted at 54 study sites in seven Asia-Pacific countries found that research participants favored being told the informed consent elements required by ethical guidelines and regulations, though the importance of each element varied [32]. For instance, in this study, risk and benefit associated with research participants were considered to be more important than the general nature or technical details of research. In a study that assessed recall and understanding of the information given to the women about a barrier contraceptive [33], while most women had an understanding about participating in research, the comprehension regarding important aspects of the study and their participation varied on key study aspects. Whereas few were unsure about ease of withdrawal from the study, the majority were aware of risks associated with use of the contraceptives, but few could actually understand the level of risk of pregnancy while using the experimental contraceptive. In a study that assessed the effects of social, cultural, and religious factors during informed consent process on a proposed HPV-serotype prevalence study in healthy pregnant women [34], awareness about health research, rights of participants, and the condition being studied was low. Besides, while most participants were skeptical and afraid of signing consent forms for research, others believed that they had an obligation to participate.

### Challenges of “understanding” research in emergency context

There is disagreement on how much understanding is necessary for informed consent for research to be valid in the emergency obstetric care context. The variation and diversity for an informed consent process in emergency contexts can be explained the complexities of an illness, ongoing therapy or meanings attached to illness by the patients. Also, how information is disclosed, how comprehension is assessed and whether viable alternative choices to participation are availed matter. For instance, use of decisional aids may improve comprehension [35–37]. Moreover, participant cognition, comprehension, decisional capacity may be affected by both severity of the illness and ongoing medication. Still, communicating equipoise is a challenging process that is easily disturbed by patients’ prior preferences and influence from friends and significant others.

Even then, participants’ views may compromise “understanding” thereby affect decision-making and voluntary choice for trial participation or choice of trial treatments. In a study that assessed participant understanding of a diabetes clinical trial in pregnancy [38], respondents were either unaware or minimized the risks of research participation, failed to recognize the nonstandard nature of the use of study drug, and consented due to feelings of satisfaction with the evidence regarding safety of the drug for their developing babies. This suggests that they deemed the study drugs to be relatively risk free. Participants may show inability to recognize minor study related risks, and presume treatments to be standard as they trusted the doctor or institution conducting the study [39]. Besides, investigators and participants may comprehend the same clinical trial information differently [40].

### The minimum understanding that participants need for participation in clinical trials

Participants need to understanding that clinical trials are conducted to address uncertainty, which include the comparative safety or effects of competing treatment alternatives. Acknowledging uncertainty is the first step to devising an effective resolution of the uncertainty through research [41, 42]. Next steps include formulating appropriate research questions and collecting appropriate data. Thus, the next element that participants need to understand is that there is some experimentation to address something that is unknown [41, 42]. Progress in therapeutics and prevention can only be feasible from willingness of individuals to voluntarily accept experimentation [43]. This necessity for experimentation is acknowledged in the Declaration of Helsinki, which states that *“medical progress is based on research which ultimately must rest in part on experimentation involving human subjects*” [2]. The third element that participants need to understand is that they may not always benefit therapeutically from research participation. If the experiment is successfully completed, investigators are in a position to learn which treatment is safer or superior [44, 45]. Informed consent to participate in research (even in emergency setting) requires understanding of the purpose, benefits, potential risks and alternatives to participation, with clear differentiation between medical care and research (much as clinical research may entail involvement in activities that relate to clinical care) [2, 10]. By asking current patients to sacrifice for the benefit of future patients, there is risk of rescinding Kant’s categorical imperative that we should treat others *“as an end in itself, never merely as a means”* [45]*.* This situation creates tension between “duty to individuals” versus “societal value” of clinical research, which necessities ensuring that an informed voluntary decision precedes enrollment for trial participation [44, 45]. While acceptable ethically from the utilitarian perspective, the knowledge gained may primarily help future patients who did not volunteer to participate in the experiment [44, 45] and the patients who actually participated may or may not benefit from the treatments being tested.

Randomization is central to a clinical trial. However, participants just need to understand the basics of the concept, rather than the details of its rationale or how it is conducted. It is well known that participants who agree to take part in a clinical trial seem to expect substantial benefit from the experimental study treatment, considering it to be novel and therefore better [21], and any information that contradicts the therapeutic misconception might be difficult for patients to assimilate let alone accept in the context of their natural hopes and anxieties [46]. Failure to understand randomization does not necessarily invalidate the informed consent process.

### Addressing “understanding” in clinical trials in emergency contexts

Informed consent requires communication of trial-related information in a way and language that can be understood by prospective participants, in order to improve comprehension, and investigators should disclose sufficient information to allow a reasonable prospective participant make a rational decision. The way clinical trial information before recruitment is presented matters, and individuals react to the same information on benefits and risks in a “predictably irrational” manner as a function of the presentation format and the context [47, 48]. That is, individuals react in significantly different ways if the same information is “framed” in terms of negative (such as morbidity, mortality, disability or poor quality of life) or beneficial outcomes (such as survival, cure and improved quality of life), or presented in terms of relative versus absolute treatment effects [47, 48]. Since, in emergency contexts, potential participants may have preferences on how and which information is disclosed, investigators have an obligation to present clinical trial information not according to their own interests, but in a neutral and transparent manner [47, 48]. This may not always correspond to the patients views in case of therapeutic optimism. However, clinical trial information has to convey the a message about the experimental nature of the research undertaking, the uncertainty about comparability of outcome effects and possibility of potential harms (all of which are at the core of the randomized clinical trial). This ensures that the process and outcome of participation is a valid method of reducing uncertainty about solution options, whereby gaining sufficient knowledge of the options can allow a reasonable selection from among them [41, 42].

Investigators should address the perception of an obligation to participate among potential research participants. The potential subject needs to know that treatment will occur with or without participation and that the subject may at any time change his or her mind about participation. Information conveyed during participant recruitment may vary considerably in content and quality [47, 48] and patients often have a poor understanding of clinical trial concepts [44, 45]. However, the voluntary nature of research participation is key, and prospective participants are should make autonomous choices [24], even if they seem irrational or illogical. Thus, in the process of seeking their voluntary consent, individuals should be free to exercise their rights to participate, including freedom to withdrawal [24].

The pervasive perception that the informed consent process may not be truly “informed” can be explained by framing effects, where it is not possible to obtain consent that is truly informed [49, 50]. Accordingly, the value of informed consent to serve as an instrument that adheres to ethical normative principle of transparent communication of benefits and risks may not be applicable (to emergency contexts) because the patients may not have stable values and preferences before enrolling in an experimental study, and these values may change over the course of participation or illness [49–51]. In addition, individuals whose consent is sought should have decisional capacity, that is, should be able to accomplish the decisional task (must have the ability to make that particular decision) [24]. “Decision-making capacity” refers to abilities related to decisions made, while “competence” refers to the state in which a patient’s decision-making capacities are sufficiently intact for the decisions to be honored regardless of who makes the determination [44]. In the emergency care context, cognition, comprehension and decisional capacity may be impaired by the illness or ongoing therapy [4–6]. Decisional capacity consists of several sub-capacities [44]: understanding (basic understanding or comprehension of the facts involved making a decision); appreciation (having insight’ into the circumstances of a given decision, that is, recognition of the nature, meaning and significance of the decision); reasoning (the ability to process the information and derive conclusions from premises, (including ability to weigh risks, benefits and evaluate potential consequences); choice (availability of choices including ability to express or communicate the intended decision or choice); values (ability to recognize and express minimally consistent and stable set of values to guide reasoning on the process of making choices). Nevertheless, uncertainty should be explicitly and transparently articulated [41], as research offers potential benefits to all, yet risk potentially occurs only to those who participate. Moreover, the conceptualization of decisional capacity as related sub-capacities is based on realization that decision-making capacity is *decision relative* [33], that is, the capacity has to be assessed relative to specific decisions in a given context at a specified time.

Transparency and accountability are therefore values that are key to “understanding” research. Investigators have a responsibility and duty to protect the rights of research subjects [2, 3]. To this end, participants who don’t comprehend the research-related information, who are incapable of making decisions (lack decisional capacity) or are unable to exercise their rights (cannot make voluntary decisions) are vulnerable to potential abuse. Rather, patients’ values are constructed during the process of elicitation, and they can easily be distorted by the “framing and elicitation effects” [51]. Consequently, the value of informed consent may lie in the process itself (a fair and transparent process) which fosters patients’ values and preferences [51]. Since efforts are made to design fair consents and consenting processes, informed consent implies that the right decisions are made, even if they may not accurately reflect patients’ preferences [50, 51].

Investigators should also recognize that participant ‘understanding’ may compete with other values such as therapeutic optimism, which relates to the value of research. Prospective participants may have predetermined decisions about whether or not to participate [52–56], even before the study information is disclosed. This may not necessarily pose a problem per se (as such decisions are indication of individuals’ autonomous interests, preferences and values), may not necessarily deter one’s ability to understand the disclosed information and nor does it limit using the disclosed information to assess benefits and risks of participation, whereby this potentially invalidates informed consent. On the contrary, it may enhance the balancing of potential risks, benefits and alternatives. Patients often make informal and quick decisions before receiving or comprehending all the relevant information presented to them during the informed consent process, without taking time to deliberate on the essential elements of the information in the informed consent [55].

Lastly, considering that many disorders in emergency obstetric care exist only as emergencies (for instance, amniotic embolism, preeclampsia, postpartum hemorrhage and obstructed labor), there is no way clinical research could otherwise be conducted if not on the respective patient populations. Other values related to justice yield equally compelling imperatives [56], and may need to be considered by research ethics committees as they deliberate on whether such clinical research in emergency contexts ought to be permitted. These include fair inclusion, fair burden sharing; fair opportunity and fair distribution of third-party risks. Other considerations include evaluation of measures to mitigate against potential harms which may occur in participants who may not fully understand the implications of clinical research participation in emergency contexts, as well as ensuring that study procedures do not unduly expose participants to potential harms. Understanding of clinical research procedures, (such as value of research, research purpose, key research questions, key study procedures, number of participants, potential study risks, potential research benefits, alternatives to research participation and possible need for compensation for research harms) should be considered in view of other equally compelling values, such as potential benefits from research participation, undue exclusion of from clinical research for patient populations in emergency contexts, and current failure to generate data that can be used to modify or improve the care of patient populations in emergency contexts.

### Implications of “understanding” for regulation of the informed consent process

Current guidelines, regulations, and best practices emphasize the need to disclose information to potential participants, with guidance on ways that should be used, rather than instead of the quality of the decision-making process [2]. Many participants may sign consent forms yet their decisions to participate are influenced by other factors. Participants’ decision-making proceeds from the informal decision (made prior to the formal informed consent process) to a formal decision (made after receiving and considering the disclosed trial information) [41]. Recognizing and understanding what factors influence the informal decision-making is crucial to improving our understanding of the patients’ decision-making and thereby understanding what participants believe is an acceptable informed consent process [41]. Whereas the informed consent document and process have value of serving as an instrument that adheres to ethical normative principle of transparent communication of benefits and risks. It may not achieve the requirement of assurance that patients to whom the information was disclosed comprehended this information, and secondly, that the participants used this information to make an informed decision about clinical research participation (in the emergency context). Unless there is a continuous process of seeking participant understanding and willingness to continue research participation, especially with assessment of whether the research participants has capacity to make valid decisions throughout the course of the research engagement, the informed consent process and document may not be valid. This is because the patients may not have stable values and preferences before enrolling in an experimental study, and these values may change over the course of participation or illness [49–51].

Decisional capacities are “decision-relative.” Therefore, capacity may change rapidly or slowly (or may remain stable) in the course of care for a patient during emergency contexts. For this reason, investigators and clinicians have an obligation to continually assess patient/participant capacity to make the required decision. Investigators have an obligation to respect the participant or patient’s decision, even if it seem wrong or irrational. Where participants change or change their values and preferences is not a sign of diminished rationality, and this has to be respected by the investigators and research ethics committees. Value swings should be expected by investigators in emergency research. Value swings among participants should be respected as a right and a reason for research participants to make decisions about continued willingness to participate in research.

Many studies on ‘understanding research’ in clinical trials focus on the content and structure of information rather than the decision-making process. However, improvements in structure and content (such as through use of shorter forms, simple language or audio-visual interventions) enhance the format for presentation of clinical trial information and may improve comprehension, but may not improve the decision-making process [57, 58]. Besides, there is insufficient evidence that decision aids improve the decision-making process [57, 58]. Whereas decision aids have potential to improve participant or patient understanding of the disclosed information, they may not necessarily improve participants’ ability to process information for participants with cognitive impairment as a result of fear, worries, illness, pain or medication, all of which may be present in clinical research in emergency contexts [59, 60]. Besides, where the decision aids have been evaluated, the focus is usually on whether they improve participants’ comprehension, and the outcomes is usually on suggestions to modify details of content information (such as amount, lay out, structure, complexity of the words) rather than on how this information may improve or may be used to improve the participants’ decision-making process.

## Conclusion

My argument is whether there is an imperative for potential clinical research participants to understand the details of the key components that make emergency clinical research ethical (value of research, research purpose, key research questions, key study procedures such as randomization, number of participants, potential study risks, potential research benefits, alternatives to research participation and possible need for compensation for research harms) is a necessity for such research to be permitted (that is, clinical research in emergency contexts). While participant ‘understanding’ of research is important, ‘understanding’ is neither necessary nor sufficient for a valid informed consent, and may compete with other values with which it needs to be considered. Prospective participants may have predetermined decisions about whether or not to participate, such that understanding may not be an important consideration for them, yet this per se does not invalidate the informed consent process.

## Data Availability

The data is available on request from the corresponding author.

## Abbreviations

ICH: International Conference on Harmonization
RCT: Randomized Clinical Trial
IRB: Institutional Review Board (IRB)
IEC: Institutional Ethics Committee
ICF: Informed Consent Form
GCP: Good Clinical Practice
QuIC: Quality of Informed Consent
